# Commentary on ‘Comparing the oncologic outcomes of local tumor destruction vs. local tumor excision vs. partial nephrectomy in T1a solid renal masses: a population-based cohort study from the SEER database’

**DOI:** 10.1097/JS9.0000000000001647

**Published:** 2024-05-15

**Authors:** Saisai Jing, Jianke Ma, Yupeng Di, Jiazhao Song

**Affiliations:** a Department of Oncology, Affiliated Cixi Hospital, Wenzhou Medical University, Cixi, Zhejiang; b Department of Radiotherapy, Air Force Medical Center, Beijing, People’s Republic of China

*Dear Editor,*

We read with great interest the recent article by Guo *et al*.^1^ entitled ‘Comparing the oncologic outcomes of local tumor destruction vs. local tumor excision vs. partial nephrectomy in T1a solid renal masses: a population-based cohort study from the SEER database’. Based on data from the Surveillance, Epidemiology, and End Results (SEER) program in the United States, they concluded that local tumor destruction (LTD) or local tumor excision/tumor resection (LTE) is more effective than partial nephrectomy (PN) in early-stage renal cancer patients. They have found that the PN has a better prognosis. This study can provide some references to the treatment options for patients with early-stage renal cancer and has far-reaching clinical significance. However, in our opinion, several issues deserve further clarification and discussion.

First, according to the patient selection methodology described by the authors in this article, the screening of this subset of patients undergoing ‘LTD’ was based on the registration of surgery codes ‘10–15’ in the SEER database. However, we found that this subset of patients had been noted in the coding menu (https://seer.cancer.gov/archive/manuals/2021/AppendixC/Surgery_Codes_Kidney_2021.pdf) as ‘No specimen sent to pathology from this surgical event.’ This suggests that the final diagnosis for this subset of patients with LTD was not clear, which we believe would interfere with the results of this paper and introduce a degree of uncertainty into the findings. We believe that it may be necessary to exclude this group of patients further and that it would be more informative to study only the impact of LTE and PN on survival.

Second, the authors balanced the baseline levels of the two groups of patients undergoing different surgical approaches by propensity score matching (PSM). In retrospective studies, this is a good statistical method to cope with baseline imbalance^2^. However, utilizing PSM in this study can only match the known factors from the SEER database, and variables that are not available from the SEER database, such as the patient’s physical condition, clinical presentation, imaging, and other factors, can also affect the survival time of the patients. This might have a potential impact on the baseline level of patients, which could lead to biased study results.

In addition, we think that a detailed description of the version of the staging ‘T1a’ in the author’s characterization of the population might be more convincing. This is because the authors chose to analyze a population of patients with a diagnosis time of 2000–2019, whereas, as far as we know, the SEER database describes T-stage with different boundaries at different diagnostic times. Patients with a diagnosis time of 2004–2015 correspond to having access to the 6th edition of the AJCC’s T-stage patients diagnosed in 2010–2015 correspond to the 7th edition of T-stage, and patients with a diagnosis time after 2018 correspond to having access to the 8th edition of T-stage^3^. However, at this time, we have difficulty clarifying the origin of the version of the T-stage chosen by the authors.

Finally, when describing the criteria for the selection of the included population, the authors lacked a description of other treatments such as radiotherapy and chemotherapy. With the advancement of radiotherapy technology, in previous studies, we have found that radiation therapy, represented by stereotactic body radiation therapy (SBRT), has better results in terms of local tumor control, toxicity, and tumor-specific survival in patients with early-stage renal cancer who did not undergo surgery in both prospective trials and retrospective analyses^4,5^. Although the description of ‘without radiotherapy and chemotherapy’ and the description of ‘unknown’ are the same as ‘without radiotherapy and chemotherapy’ in the SEER database, the authors did not describe the criteria for the selection of the included population. Although the description of ‘non-radiotherapy and non-chemotherapy’ in the SEER database is the same record as ‘unknown,’ the radiotherapy and chemotherapy that were received were available, and the order of treatment (whether preoperative or postoperative radiotherapy was given) could also be obtained from the SEER database^6^. In conclusion, we believe that in the cohort of patients in this study, there may still be non-surgical antitumor treatments such as radiotherapy or chemotherapy that interfere to some extent with the survival benefit of surgery.

All in all, we highly agree with the clinical significance of this study and analysis conducted by the authors, which fills the research gap in the comparison of the benefits of early renal surgical modalities and is worthy of further prospective multicenter studies. At the same time, we’d like to suggest to the authors that perhaps it could be done to improve the reliability and clinical value of the study, by further improving the methodology as well as embellishing the images in future studies.

## Ethical approval

None.

## Consent

None.

## Sources of funding

This work was supported by grants from the Project of NINGBO Leading Medical & Health Discipline (Project Number: 2022-X22).

## Author contribution

J.S., Y.D., and S.J.: study concept or design; S.J. and J.M.: writing the paper; J.S., Y.D., and S.J.: review and revise.

## Conflicts of interest disclosure

There are no conflicts of interest.

## Research registration unique identifying number (UIN)

None.

## Guarantor

Jiazhao Song, MD; Yupeng Di, MD; and Saisai Jing, MD.

## Data availability statement

All data generated or analyzed during this study are included in this article, only from ‘Comparing the oncologic outcomes of local tumor destruction vs. local tumor excision vs. partial nephrectomy in T1a solid renal masses: a population-based cohort study from the SEER database.’

## Provenance and peer review

None

## References

[1] Guo R-Q Peng J-Z Sun J . Comparing the oncologic outcomes of local tumor destruction vs. local tumor excision vs. partial nephrectomy in T1a solid renal masses: a population-based cohort study from the SEER database. Int J Surg 2024; Apr 10 [Epub ahead of print] 10.1097/JS9.0000000000001465 PMC11325914 38597382

[2] Austin PC . An introduction to propensity score methods for reducing the effects of confounding in observational studies. Multivar Behav Res 2011;46:399–424. 10.1080/00273171.2011.568786 PMC3144483 21818162

[3] National Cancer Institute. Surveillance, Epidemiology, and End Results Program. SEER*Stat Software, version 8.4.3 www.seer.cancer.gov/seerstat

[4] Siva S Bressel M Sidhom M . FASTRACK II Investigator Group . Stereotactic ablative body radiotherapy for primary kidney cancer (TROG 15.03 FASTRACK II): a non-randomised phase 2 trial. Lancet Oncol 2024;25:308–316. 38423047 10.1016/S1470-2045(24)00020-2

[5] Yamamoto T Kawasaki Y Umezawa R . Stereotactic body radiotherapy for kidney cancer: a 10-year experience from a single institute. J Radiat Res 2021;62:533–539. 33866363 10.1093/jrr/rrab031 PMC8127673

[6] Surveillance, Epidemiology, and End Results (SEER) Program (www.seer.cancer.gov) SEER*Stat Database: Incidence - SEER Research Data, 17 Registries, Nov 2022 Sub (2000–2020) - Linked To County Attributes - Time Dependent (1990–2021) Income/Rurality, 1969–2021 Counties, National Cancer Institute, DCCPS, Surveillance Research Program, released April 2023, based on the November 2022 submission.

